# COVID-19 Contact Tracing in Two Counties — North Carolina, June–July 2020

**DOI:** 10.15585/mmwr.mm6938e3

**Published:** 2020-09-25

**Authors:** R. Ryan Lash, Catherine V. Donovan, Aaron T. Fleischauer, Zack S. Moore, Gibbie Harris, Susan Hayes, Meg Sullivan, April Wilburn, Jonathan Ong, Dana Wright, Raynard Washington, Amy Pulliam, Brittany Byers, Heather P. McLaughlin, Emilio Dirlikov, Dale A. Rose, Henry T. Walke, Margaret A. Honein, Contact Tracing Assessment Team, Patrick K. Moonan, John E. Oeltmann, Contact Tracing Assessment Team, Sean Griffing, Tanya Williams, Carolina Luna-Pinto, Raul Romaguera, Phoebe Thorpe, Julie Villanueva, Kyle Bernstein, Rachel Woodruff, Mary McFarlane, Eric Pevzner, Andrew Cressman, North Carolina, Victoria Mobley, North Carolina, Erika Samoff

**Affiliations:** ^1^Epidemic Intelligence Service, CDC; ^2^CDC COVID-19 Response Team; ^3^North Carolina Department of Health and Human Services, Raleigh, North Carolina; ^4^Career Epidemiology Field Officer Program, CDC; ^5^Mecklenburg County Public Health, Charlotte, North Carolina; ^6^Randolph County Public Health, Asheboro, North Carolina.; Contact Tracing Assessment Team, CDC; Contact Tracing Assessment Team, CDC; Contact Tracing Assessment Team, CDC; Contact Tracing Assessment Team, CDC; Contact Tracing Assessment Team, CDC; Contact Tracing Assessment Team, CDC; Contact Tracing Assessment Team, CDC; Contact Tracing Assessment Team, CDC; Contact Tracing Assessment Team, CDC; Contact Tracing Assessment Team, CDC; Contact Tracing Team; Contact Tracing Team; Contact Tracing Team; North Carolina.

*On September 22, 2020, this report was posted online as an *MMWR *Early Release.*

Contact tracing is a strategy implemented to minimize the spread of communicable diseases (*1*,*2*). Prompt contact tracing, testing, and self-quarantine can reduce the transmission of SARS-CoV-2, the virus that causes coronavirus disease 2019 (COVID-19) (*3*,*4*). Community engagement is important to encourage participation in and cooperation with SARS-CoV-2 contact tracing (*5*). Substantial investments have been made to scale up contact tracing for COVID-19 in the United States. During June 1–July 12, 2020, the incidence of COVID-19 cases in North Carolina increased 183%, from seven to 19 per 100,000 persons per day* (*6*). To assess local COVID-19 contact tracing implementation, data from two counties in North Carolina were analyzed during a period of high incidence. Health department staff members investigated 5,514 (77%) persons with COVID-19 in Mecklenburg County and 584 (99%) in Randolph Counties. No contacts were reported for 48% of cases in Mecklenburg and for 35% in Randolph. Among contacts provided, 25% in Mecklenburg and 48% in Randolph could not be reached by telephone and were classified as nonresponsive after at least one attempt on 3 consecutive days of failed attempts. The median interval from specimen collection from the index patient to notification of identified contacts was 6 days in both counties. Despite aggressive efforts by health department staff members to perform case investigations and contact tracing, many persons with COVID-19 did not report contacts, and many contacts were not reached. These findings indicate that improved timeliness of contact tracing, community engagement, and increased use of community-wide mitigation are needed to interrupt SARS-CoV-2 transmission.

Routinely collected case investigation and contact tracing data from June 1–30, 2020, for Mecklenburg, and from June 15–July 12, 2020, for Randolph counties were analyzed. Case investigations were conducted for persons with laboratory-confirmed COVID-19, including the elicitation of persons potentially exposed to the index patient (*3*). Contact tracing was performed for persons identified as close contacts and included inquiry about COVID-19–compatible symptoms^†^ and instructions to self-quarantine for 14 days since last exposure (*3*). Health department staff members monitored contacts for new-onset symptoms. SARS-CoV-2 diagnostic testing was encouraged for all close contacts (*3*). Persons with COVID-19 and contacts were classified as lost to follow-up if they did not respond after three failed attempts to contact them at different times on consecutive days or if contact information was missing or invalid. COVID-19 case-based surveillance data are maintained within the North Carolina Electronic Disease Surveillance System; contact-based information is maintained within the state-supported COVID-19 Community Team Outreach tool. Mecklenburg County uses a commercial information management system, HealthSpace Data Systems Ltd., for case management. This activity was determined to be public health surveillance as defined in 45 CFR 46.102(l).^§^

Mecklenburg County has an estimated population of 1,110,356 persons (*6*) most of whom live in the city of Charlotte. In June, Mecklenburg County conducted 61,979 SARS-CoV-2 tests resulting in 8,097 (13%) positive results. Among these, 7,116 (88%) were confirmed as new COVID-19 cases in county residents (Table); the remaining were in residents of other jurisdictions or retests. During the assessment period, an average of 24 cases per 100,000 persons occurred per day. The median interval from specimen collection to reported results was 2 days (range = 0–29 days); 23% (1,602 of 7,116) of laboratory-confirmed cases were lost to follow-up. Overall, 5,514 (77%) persons with positive test results were reached for case investigation and elicitation of contacts; the median interval from specimen collection to case investigation was 4 days (range = 0–38 days). Among COVID-19 patients interviewed, 2,624 (48%) reported no contacts. Among those who did report contacts, 13,401 contacts were named (average contacts per case = 4.6). The median interval from case investigation to contact notification was 1 day (range = 0–25 days). Among reported contacts, 3,331 (25%) were lost to follow-up. An additional 255 (2%) contacts were reached and counseled to quarantine but declined monitoring by the health department. Therefore, 9,815 (73%) reported contacts were reached, assessed for current symptoms, counseled to quarantine, and monitored daily by the health department. The median interval between specimen collection and contact notification was 6 days (range = 1–38 days). The total number of contacts tested was not available because contact status was not a required variable on the laboratory requisition form; however, during follow-up, 137 contacts had laboratory-confirmed COVID-19.

**TABLE:** COVID-19 contact tracing metrics in two counties — North Carolina, June–July 2020

Metrics	Mecklenburg County*	Randolph County*
**No. of specimens tested**	61,979	6,292
**Case investigation, no. (%)**		
Positive laboratory reports received^†^	8,097 (13)	707 (11)
Laboratory-confirmed COVID-19 cases	7,116 (88)^§^	589 (83)^§^
Laboratory-confirmed COVID-19 cases lost to follow-up	1,602 (23)^¶^	5 (1)**
Laboratory-confirmed COVID-19 cases with initial investigation	5,514 (77)	584 (99)
Laboratory-confirmed COVID-19 cases with initial investigation with no contacts named	2,624 (48)	202 (35)
Laboratory-confirmed COVID-19 cases with named contacts	2,890 (52)	382 (66)
**Contact tracing, no. (%)**		
No. of Identified contacts	13,401^††^	1,146
Identified contacts lost to follow-up	3,331 (25)^¶^	544 (47)^¶^
Identified contacts opted out of health department daily monitoring	255 (2)	50 (4)
Identified contacts who agreed to self-quarantine and 14-day monitoring	9,815 (73)^§§^	552 (48)^§§^
Identified contacts who agreed to self-quarantine and subsequently had a positive test result	137^¶¶^	69***
**Time intervals, no. of days (range)**		
From specimen collection to reported results	2 (0–29)	3 (0–15)
From specimen collection to case investigation	4 (0–38)	3 (0–36)
From case investigation to contact notification	1 (0–25)	3 (0–26)
From specimen collection to contact notification and presumed start of quarantine	6 (1–38)	6 (0–58)

**Abbreviation:** COVID-19 = coronavirus disease 2019.

* In some cases, column percentages within a category might not sum to 100% because of rounding.

^†^ Difference between positive laboratory reports received and laboratory-confirmed cases (981 in Mecklenburg County and 118 in Randolph County) reflects testing of residents from other jurisdictions or repeat testing.

^§^ Cases in county residents; the remaining cases were in residents of other jurisdictions or retests.

^¶^ Could not be reached via phone after 3 consecutive days of failed attempts, or if contact information was missing or invalid.

** Could not be reached via phone after 3 consecutive days of failed attempts and a visit by local law enforcement to the residential address provided, or if contact information was missing or invalid.

^††^ Does not include contacts identified during investigations of congregate settings or large workplace investigations.

^§§^ Contacts were monitored by the health department.

^¶¶^ The total number of contacts who volunteered to be tested is unknown.

*** In total, 293 contacts volunteered to be tested.

Randolph County has an estimated population of 143,667 (*6*). During June 15–July 12, Randolph County conducted 6,292 SARS-CoV-2 tests, resulting in 707 positive results. Among these, 589 (83%) were confirmed as new COVID-19 cases among county residents (Table). During the assessment period, an average of 15 cases per 100,000 persons occurred per day. The median interval from specimen collection to reported results was 3 days (range = 0–15 days). Among persons with reported cases, 584 (99%) were reached for case investigation and elicitation of contacts; five (1%) were lost to follow-up, even after dispatching law enforcement to the residential address provided. The median interval from specimen collection date to case investigation date was 3 days (range = 0–36 days). Among COVID-19 patients interviewed, 202 (35%) reported no contacts. Among those who did report contacts, 1,146 were named (average = three contacts per case). An increasing trend in the percentage of cases not reporting contacts was observed, from 26% during week 1 (June 1–7) to 48% during week 4 (June 22–28) of the assessment. The median interval from case investigation to contact notification was 3 days (range = 0–26 days). Among 1,146 reported contacts, 544 (47%) were lost to follow-up. An additional 50 (4%) contacts were reached and counseled to quarantine but declined monitoring by the health department. Thus, 552 (48%) reported contacts were reached, assessed for current symptoms, counseled to quarantine, and monitored daily. The median duration between specimen collection and contact notification was 6 days (range = 0–58 days). A total of 293 (53%) contacts who started quarantine received a SARS-CoV-2 test during follow-up; 69 (24%) results were positive.

## Discussion

Health department staff members began investigation of 77% to 99% of new COVID-19 cases within a median of 3–4 days from specimen collection. However, 35% (Randolph County) to 48% (Mecklenburg County) of patients with COVID-19 did not report contacts. This proportion is high relative to proportions noted for other infectious diseases before the COVID-19 pandemic in the United States (*1*,*7*). There are a few probable reasons for this. First, limiting contact tracing to a telephone conversation might have inhibited the ability of public health workers to establish a rapport and elicit contacts. Second, persons with COVID-19 might have sought to avoid subjecting their contacts to quarantine control measures, including potential loss of work and related economic consequences. Despite efforts to reach all elicited contacts, one quarter of contacts in Mecklenburg and nearly one half in Randolph County were not reachable. Contacts might have been reluctant to answer phone calls from unknown numbers; 2%–4% who were reached declined health department monitoring. Finally, the high volume of work might have contributed to staff members’ ability to trace contacts (*8*).

These results are comparable to COVID-19 data reported from other U.S. states. Data from Maryland^¶^ and New Jersey** indicate that 50% and 52% of reported cases, respectively, reported no contacts. Similarly, the proportion of contacts reached in Maryland (50%) and New Jersey (54%) were comparable. The relatively low participation and cooperation with contact tracing suggests a lack of community support and engagement with contact tracing. This, coupled with delays in testing results are contributing to ongoing transmission. To increase the timeliness and completeness of contact tracing, the North Carolina Department of Health and Human Services hired additional staff members to support local health departments, enhanced data systems, and pursued new technologies such as a single statewide caller identification number.

The findings in this report are subject to at least three limitations. First, both study locations were experiencing high and increasing COVID-19 incidence during the review period; high caseload volumes stress the system and can result in delays for testing, cases investigation, and contact tracing. Second, data drawn from county health department information systems are self-reported by patients or contacts, which could affect data validity. For example, a social desirability bias could have led to the underreporting of contacts because it is understood that contact with more persons increases risk for transmission. Finally, information about why so many persons with COVID-19 reported no contacts and why so many contacts were not reached was not available. This failure to comply with public health recommendations might reflect the various, and at times conflicting, messages about the importance of COVID-19 mitigations strategies^††^ (*9*).

This assessment revealed that, although these two county health departments investigated the majority of index cases, a high proportion of persons with COVID-19 did not report contacts, many contacts were not reached, and the time needed to notify contacts likely reduced the impact of contact tracing as a mitigation strategy. Improved timeliness of contact tracing, community engagement, and community-wide mitigation are needed to interrupt SARS-CoV-2 transmission (*4*,*6*).

**Summary**

**What is already known about this topic?**

Successful SARS-CoV-2 contact tracing requires timeliness and community engagement to encourage participation and cooperation.

**What is added by this report?**

During periods of high COVID-19 incidence in North Carolina, 48% of COVID-19 patients reported no contacts, and 25% of contacts were not reached in Mecklenburg County. In Randolph County, 35% of COVID-19 patients reported no contacts, and 48% of contacts were not reached. Median interval from index patient specimen collection to contact notification was 6 days.

**What are the implications for public health practice?**

Despite aggressive efforts by health departments, many COVID-19 patients do not report contacts, and many contacts cannot be reached. Improved timeliness of contact tracing, community engagement, and community-wide mitigation are needed to reduce SARS-CoV-2 transmission.

## Footnotes

* https://covid19.ncdhhs.gov/dashboard/cases.

† https://www.cdc.gov/coronavirus/2019-ncov/symptoms-testing/symptoms.html.

§ U.S. Department of Health and Human Services, Title 45 Code of Federal Regulations 46, Protection of Human Subjects.

¶ https://coronavirus.maryland.gov/pages/contact-tracing.

** https://www.nj.gov/health/cd/topics/covid2019_dashboard.shtml.

†† https://www.theguardian.com/us-news/2020/jun/28/north-carolina-coronavirus-reopening-cases-businesses.

## References

[1] Rothenberg RB, McElroy PD, Wilce MA, Muth SQ. Contact tracing: comparing the approaches for sexually transmitted diseases and tuberculosis. Int J Tuberc Lung Dis 2003;7(Suppl 3):S342–8. 14677820

[2] Bell BP, Damon IK, Jernigan DB, Overview, control strategies, and lessons learned in the CDC response to the 2014–2016 Ebola epidemic. MMWR Suppl 2016;65(No. Suppl 3). 10.15585/mmwr.su6503a2 27389903

[3] CDC. Coronavirus disease 2019 (COVID-19): interim guidance on developing a COVID-19 case investigation and contact tracing plan. Atlanta, GA: US Department of Health and Human Services, CDC; 2020. https://www.cdc.gov/coronavirus/2019-ncov/php/contact-tracing/contact-tracing-plan/overview.html

[4] Kretzschmar ME, Rozhnova G, Bootsma MCJ, van Boven M, van de Wijgert JHHM, Bonten MJM. Impact of delays on effectiveness of contact tracing strategies for COVID-19: a modelling study. Lancet Public Health 2020;5:e452–9. 10.1016/S2468-2667(20)30157-2 32682487 PMC7365652

[5] National Academies of Sciences, Engineering, and Medicine. Encouraging participation and cooperation in contact tracing: lessons from survey research. Washington, DC: The National Academies Press; 2020.

[6] US Census Bureau. Population and housing unit estimates tables, vintage year 2019. Washington DC: US Department of Commerce, US Census Bureau; 2020. https://www.census.gov/programs-surveys/popest/data/tables.html

[7] Reichler MR, Reves R, Bur S, ; Contact Investigation Study Group. Evaluation of investigations conducted to detect and prevent transmission of tuberculosis. JAMA 2002;287:991–5. 10.1001/jama.287.8.991 11866646

[8] Clark E, Chiao EY, Amirian ES. Why contact tracing efforts have failed to curb COVID-19 transmission in much of the U.S. Clin Infect Dis 2020. Epub August 6, 2020. 10.1093/cid/ciaa1155 32761123 PMC7454341

[9] Altman D. Understanding the US failure on coronavirus—an essay by Drew Altman. BMJ 2020;370:m3417. 10.1136/bmj.m3417 32928876

